# Poster Session II - A272 NON-ANTI-TNF ADVANCED THERAPEIS FOR THE MANAGEMENT OF ORAL CROHN’S DISEASE: A CASE SERIES

**DOI:** 10.1093/jcag/gwaf042.272

**Published:** 2026-02-13

**Authors:** K Alseiari, R Sedano, A Wilson

**Affiliations:** Western University Schulich School of Medicine & Dentistry, London, ON, Canada; Western University Schulich School of Medicine & Dentistry, London, ON, Canada; Western University Schulich School of Medicine & Dentistry, London, ON, Canada

## Abstract

**Background:**

Oral Crohn’s disease (OCD) is a rare and debilitating extraintestinal manifestation characterized by painful ulcers, mucosal edema, and granulomatous inflammation of the face and/or mouth. Evidence guiding management remains limited, particularly for patients with refractory disease or anti-tumor necrosis factor-a (anti-TNF) therapy failure.

**Aims:**

Given the limited existing literature, we aimed to describe the use and effects of non-anti-TNF based therapies in OCD.

**Methods:**

Patients with histologically confirmed OCD treated with non-anti-TNF advanced therapies seen at two academic hospitals affiliated with Western University were identified. Data, including demographics, disease duration, treatment regimens, and longitudinal oral disease outcomes, were collected and summarized.

**Results:**

Three female patients with histologically-confirmed OCD were identified. The median disease duration was 33 years (interquartile range, IQR=14 years). All had concomitant and severe intestinal disease at presentation, requiring a minimum of 2 surgeries over their disease course with a median time to first CD-related surgery of 2 years (IQR=5 years). All had anti-TNF exposure with secondary loss of response. Two of 3 patients experienced major anti-TNF adverse events (paradoxical psoriasis, septic arthritis) while on therapy.

All patients had significant OCD involving, at minimum, the tongue, palate, buccae, with significant symptomatic burden. Median time to OCD diagnosis was 32 years (IQR=6.5 years). One patient developed OCD within 15 months of anti-TNF discontinuation, while two patients developed OCD while on treatment with an anti-TNF.

Following the introduction of non-anti-TNF advanced therapies, all patients achieved complete resolution of OCD lesions with a median time to resolution of 6 months (IQR=5months). One patient achieved complete remission of oral lesions on high-dose vedolizumab. The second patient demonstrated full oral mucosal resolution on standard-dose risankizumab, and the third experienced a dose-dependent clinical response to upadacitinib, with recurrence of her oral disease upon dose de-escalation and recovery after re-escalation. All patients received concurrent oral dexamethasone mouth rinses, with only one patient receiving concurrent systemic glucocorticoid treatment (budesonide). No significant adverse events were observed during a one-year follow-up period.

**Conclusions:**

This case series underscores a possible role for non-anti-TNF advanced therapies for the management of OCD. Herein, we highlight the successful use of α4β7-integrin blockade, IL-23 inhibition, and JAK inhibition for the management of this rare, but debilitating CD phenotype. These findings are hypothesis-generating. Further prospective studies are warranted to clarify long-term efficacy, durability of remission, and optimal treatment sequencing for non-anti-TNF therapies in OCD.

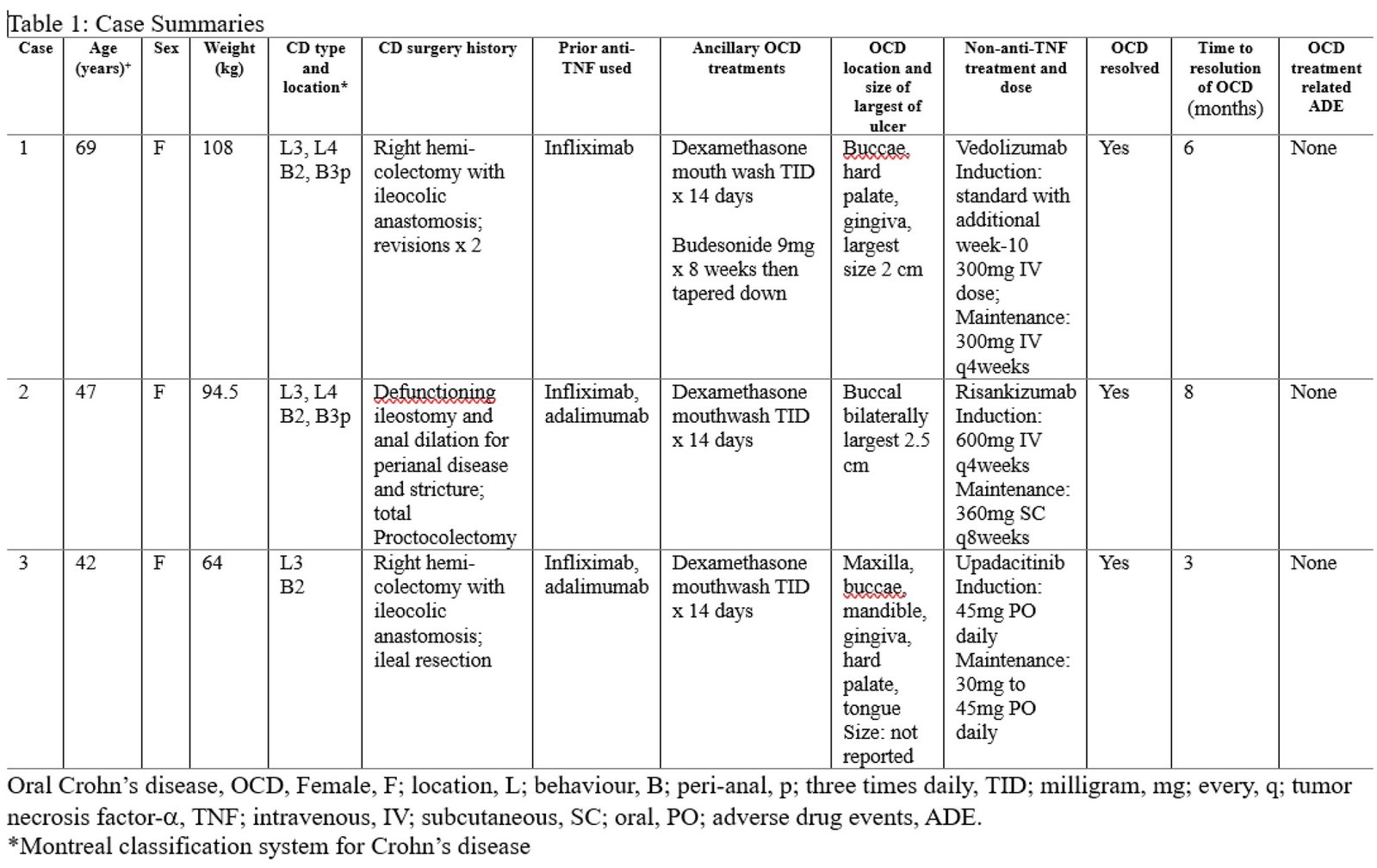

**Funding Agencies:**

None

